# Biomechanical Value of a Protective Proximal Humeral Cerclage in Reverse Total Shoulder Arthroplasty

**DOI:** 10.3390/jcm10194600

**Published:** 2021-10-06

**Authors:** Philipp A. Michel, J. Christoph Katthagen, Benedikt Schliemann, Sina Wilkens, Andre Frank, Lukas F. Heilmann, Felix Dyrna, Michael J. Raschke

**Affiliations:** Department of Trauma, Hand and Reconstructive Surgery, University Hospital Muenster, 48149 Muenster, Germany; Christoph.Katthagen@ukmuenster.de (J.C.K.); Benedikt.Schliemann@ukmuenster.de (B.S.); sina.wilkens@googlemail.com (S.W.); Andre.Frank@ukmuenster.de (A.F.); Lukas.Heilmann@ukmuenster.de (L.F.H.); Felix.Dyrna@ukmuenster.de (F.D.); Michael.Raschke@ukmuenster.de (M.J.R.)

**Keywords:** proximal humeral fracture, reverse shoulder arthroplasty, cerclage

## Abstract

Reverse shoulder arthroplasty (RSA) is a commonly performed salvage procedure for failed proximal humeral fracture fixation. The rate of intraoperative periprosthetic fractures is higher compared to primary RSA. The goal of this study was to investigate the biomechanical value of a protective cerclage during stem impaction in a revision surgery setting. Twenty-eight fresh-frozen human humeri were used to assess different configurations for steel wire and FiberTape cerclages. A custom-built biomechanical test setup simulated the mallet strikes during the stem impaction process with the Univers Revers prothesis stem. The mallet energy until the occurrence of a first crack was not different between groups. The total energy until progression of the fracture distally to the cerclage was significantly higher in the cerclage groups compared to the native humerus (9.5 J vs. 3.5 J, respectively; *p* = 0.0125). There was no difference between the steel wire and FiberTape groups (11.4 J vs. 8.6 J, respectively; *p* = 0.2695). All fractures were located at the concave side of the stem at the metaphyseal calcar region. This study demonstrates that a protective cerclage can successfully delay the occurrence of a fracture during stem impaction in reverse shoulder arthroplasty. A FiberTape cerclage is biomechanically equally efficient compared to a steel wire cerclage.

## 1. Introduction

Proximal humeral fractures (PHF) are common, especially in the elderly population. Epidemiological studies show an overall incidence of 50/100,000 for males and 175/100,000 for females [1,2,3]. Those numbers rise to over 397/100,000 for females aged between 75 and 84 years. Over the last 20 years, the total count of PHFs has increased by 137% for males and 39% for females in this age group [4].

It is still under debate which type of treatment (non-operative, open reduction and internal fixation (ORIF) or reverse total shoulder arthroplasty (RSA)) fits best for the individual patient and fracture. For elderly patients with a comminuted three- or four-part fracture, the current literature appears to favour RSA [5,6,7]. Due to high complication rates with varus loss of reduction, articular screw perforation, and humeral head necrosis, the conversion from a failed fracture fixation to RSA is a commonly performed salvage procedure. Multiple retrospective studies show that this successfully improves shoulder function and pain scores [8,9,10,11,12]. However, the number of revision surgeries following RSA for failed ORIF is significantly higher compared to primary arthroplasty (4.4% vs. 19.4%) [13,14,15].

The intraoperative complication rate for primary RSA has been reported to range between 1.5% and 2.5% and might even be slightly higher in trauma patients. One of the most common problems is a periprosthetic fracture during humeral shaft preparation and stem impaction [16,17,18]. For revision RSA, the rate of humeral fractures can increase up to 16% due to the necessary component removal. Additionally, the remaining shaft is weakened by the bi-cortical screw holes after plate fixation [19]. For fracture fixation, a steel cerclage wire, cable cerclage, or suture cerclage is commonly placed around at the proximal humeral shaft. A recent clinical study from Eyberg et al. showed that a suture cerclage is safe and effective for humeral fixation in shoulder arthroplasty. The authors used a No. 5 FiberWire (Arthrex, Naples, FL, USA) for 1–4 suture cerclages for fixation of humeral osteotomies and periprosthetic fractures. All cases demonstrated radiographic healing and no complications related to the cerclage fixation were observed [20]. Similar results were obtained by Kriechling et al. The authors reviewed 39 cases of intraoperative calcar fractures, which were treated with suture and cable cerclages, or without intervention [21]. A recent biomechanical study compared a stainless-steel wire cerclage (SSWC) with single- (SLTC) and double-looped (DLTC) tensionable suture-based cerclages for stabilization of humeral osteotomies during shoulder arthroplasty. They found higher failure loads and lower gap displacement for the DLTC group compared with the SSWC group [22].

Although this demonstrates effectiveness, little is known about the effect of a protective cerclage at the proximal humeral shaft in the context of revision RSA. Currently, there is a shift away from cemented arthroplasty towards cementless prosthetic replacement. Since cementless replacement relies on a press-fit type of fixation of the prosthetic stem, increased stability is of great interest. Biomechanical data indicate that protective cerclages may prevent crack propagation and increase the rotational- and total energy to failure in press-fit femoral implants [23,24].

**Figure 1:**
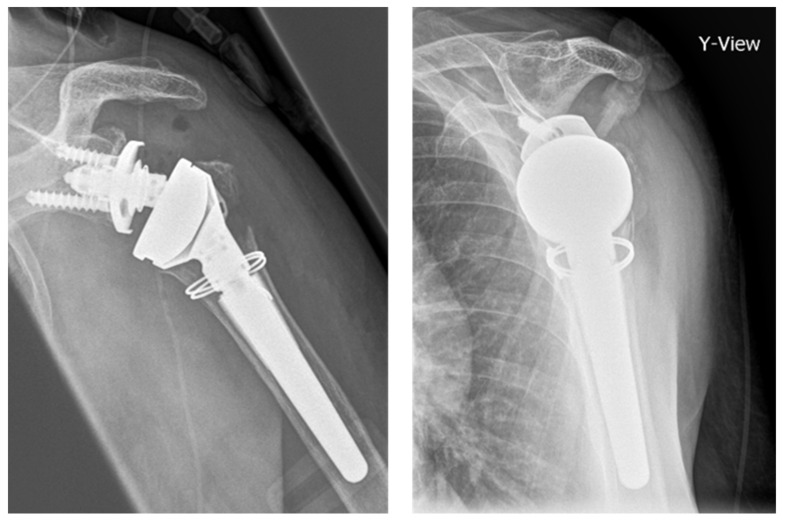
X-rays of the left shoulder of an 82 year-old patient after implantation of a modular, non-cemented proximal humeral fracture prosthesis (RSA) with protective cerclage (1.5 mm steel wire) at the metaphyseal shaft.

In patients with PHF, osteoporosis and a low cortical index with a thin cortical shell are common factors complicating the stem preparation process (Figure 1). In RSA for failed fracture fixation, the screw-holes of the removed implant further decrease humeral stability. To date, little is known about the biomechanical impact of a protective humeral shaft cerclage for revision RSA after locked plating of PHFs. Furthermore, it remains unclear if the cerclage material influences the biomechanical stability.

First, we hypothesized that a protective cerclage would delay the occurrence of an intraoperative periprosthetic fracture compared to the native humeral situation. Second, we hypothesized that the FiberTape cerclage would be biomechanically equally efficient compared to the steel wire cerclage. Third, we hypothesized that the tensioning procedure of the FiberTape cerclage would not negatively affect the biomechanical stability.

## 2. Materials and Methods

### 2.1. Specimens

For the study, a total of 28 fresh-frozen human humeri were used. Institutional review board approval was obtained prior to the study (IRB No. 2014–421-f-N). The mean age of the donors was 78.1 years (range 62–93 years) including 12 males and 16 females. The bone mineral density (BMD) was assessed by quantitative computer tomography (CT) at the center of the humeral head. The mean BMD of all specimens was 85.4 mg/cm^3^ (range 29.8–168.8 mg/cm^3^), indicating osteoporotic bone quality [25,26,27].

All humeri had previously been tested in a biomechanical model investigating different plating options for PHFs [28]. This included a gap osteotomy of 10 mm below the anatomical neck and fixation with a 3-hole PHILOS-plate (DePuy Synthes, Umkirch, Germany) with 1 non-locking- and 2 locking screws (3.5 mm) to the humeral shaft of all specimens. After axial testing, the implant components were carefully removed and the remaining soft tissue was stripped. The specimens were potted in Polymethylmethacrylat (PMMA—Technovit, Kulzer, Werheim, Germany) with a minimum remaining shaft length of 9 cm outside the potting material. Since all specimens had plate fixation of a PHF, conversion from failed ORIF to RSA could easily be simulated with this setup [12]. The remaining shaft was stored in a double vacuum-sealed plastic bag at −20 °C. Prior to preparation and testing, the specimens were thawed at room temperature for 12 h [29]. Before testing, all specimens were inspected by two independent investigators for visible cracks or fractures due to previous testing. No specimens had to be excluded.

### 2.2. Cortical Thickness Measurement

The cortical thickness was measured with the deltoid tuberosity index (DTI) using anterior–posterior (a.-p.). X-rays were taken of the specimens according to the method described by Spross et al. [30]. The authors found that a DTI consistently lower than 1.4 indicates a low local BMD of the proximal humerus. Of the 28 humeri examined, 25 had a DTI ≤ 1.4 (mean 1.33, range 1.18–1.54). There was no significant difference between the groups (Table 1).

### 2.3. Cerclage Fixation

**Figure 2:**
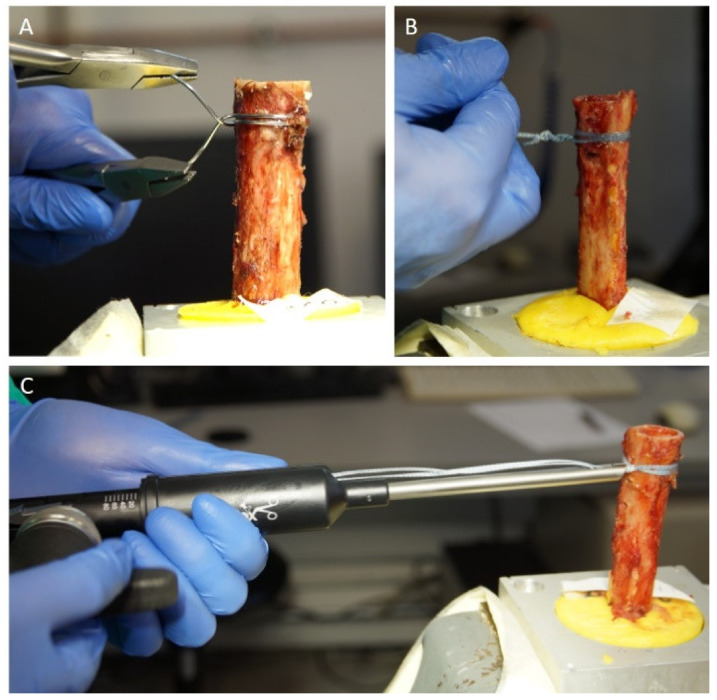
(**A**): Application of the 1.5 mm steel cerclage wire in group 1 (steel wire). (**B**): Hand-knotting of the FiberTape cerclage in group 3 (FT-hand). (**C**): Tensioning of the FiberTape cerclage system in group 2 (FT-tension).

The humeri were randomized into 4 different groups. All cerclages were placed 2 cm distal to the humeral osteotomy and were performed by a single experienced trauma surgeon. A double looped 1.5 mm steel cerclage wire was carefully tightened and locked according to standardized protocols for group 1 (steel wire) [31]. In group 2 (FT-tension) a double looped 2 mm FiberTape cerclage suture (AR-7267-1, Arthrex) was used and a tension of 80 pounds was applied with the cerclage tensioner set (AR-7800S, Arthrex). The cerclage suture was locked by 7 half-hitches (surgeons knot [32]) according to the manufacturer’s guidelines. In group 3 (FT-hand), a double-looped 2 mm FiberTape (AR-7237, Arthrex) was tightened by hand and locked with 7 half-hitches similar to group 2. Group 4 (native) served as a control group without any cerclage treatment (Figure 2).

### 2.4. Biomechanical Testing

**Figure 3:**
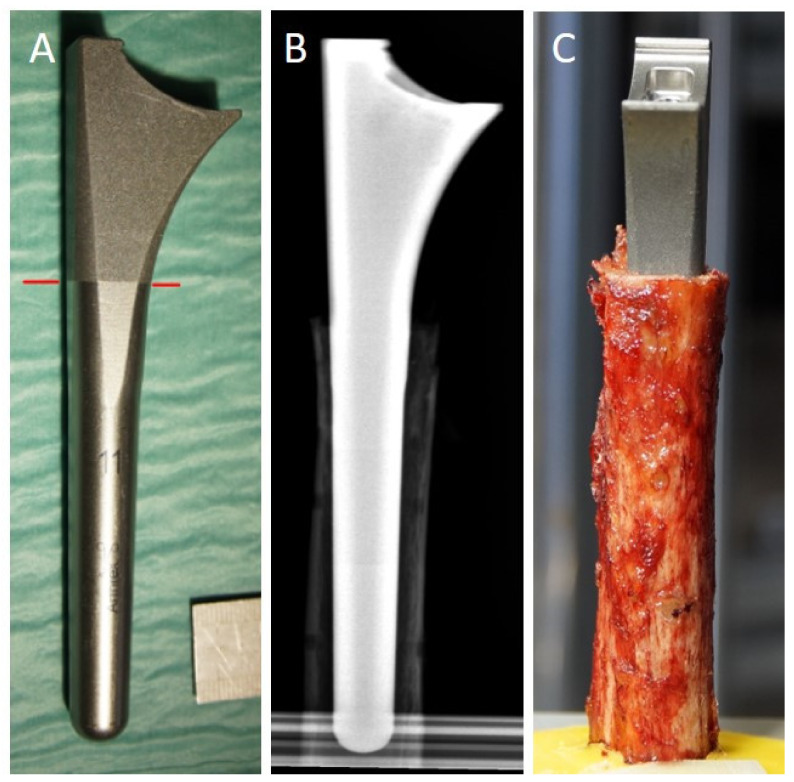
(**A**) Univers Revers stem (size 11), red line marks the junction between dia- and meta-physis. (**B**) X-ray prior to biomechanical testing confirming the correct stem size in relation to the humeral shaft. (**C**) Clinical picture prior to the start of testing confirming the correct position of the dia-/meta-physal junction in relation to the humeral shaft entrance.

For biomechanical testing, we used the Univers Revers total shoulder system (Arthrex). The choice of the correct stem size for the respective specimen was performed carefully. According to clinical pre-operative arthroplasty planning, we measured the intramedullary diameter at the humeral shaft entrance and a single experienced trauma surgeon selected the appropriate stem size. Stem sizes from 6 to 15 mm were available. The humeral shaft was then reamed according to standardized clinical procedures. When the stem’s junction from dia- to meta-physis overlapped with the osteotomy height and the stem would not subside any further into the humerus without mallet strikes, the stem size was considered to be correct (Figure 3).

The majority of the studies we screened for methodological reasons for this study utilized a biomechanical testing machine setup with a constantly applied forward motion of the stem. In our opinion, this does not optimally simulate the surgical stem impaction process with mallet strikes. We created a new standardized biomechanical test-setup to simulate the mallet strike on the prothesis stem during the impaction procedure.

**Figure 4:**
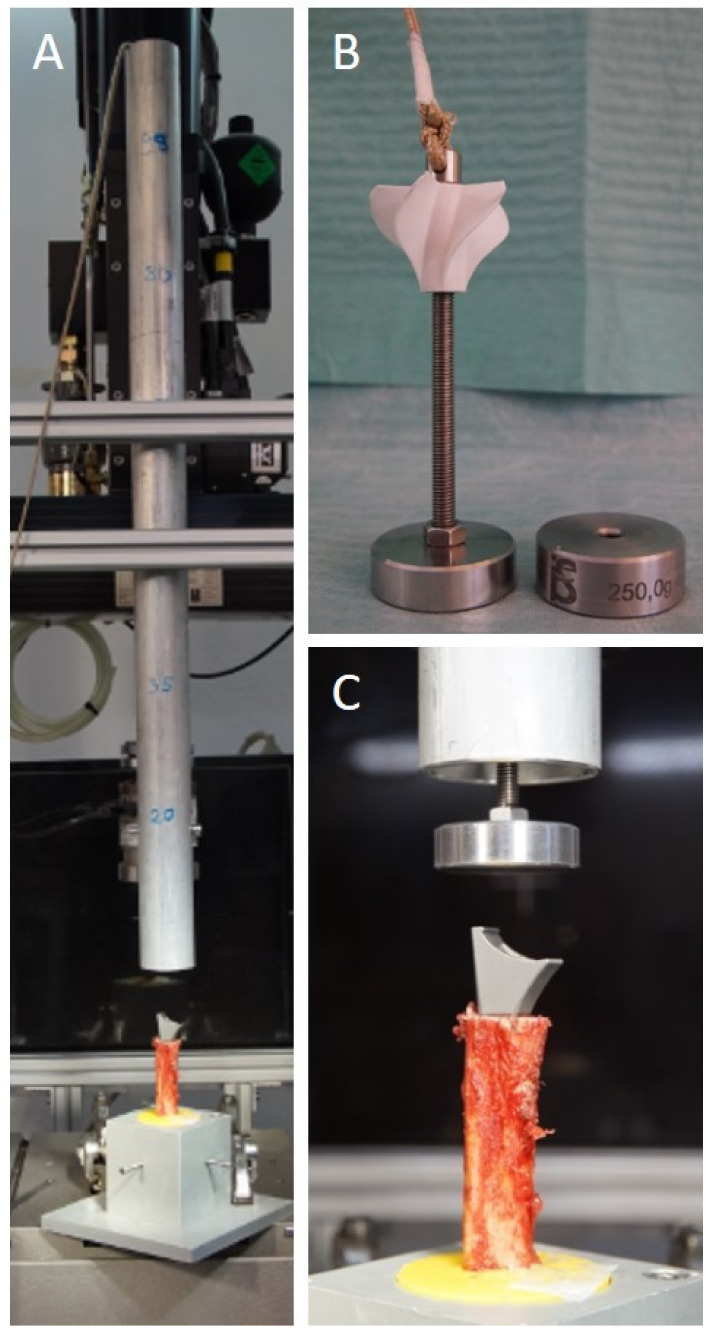
(**A**) Complete biomechanical test-setup with the downpipe placed vertically above the specimen. (**B**) 250 g (+additional 250 g) weight placed inside the tube can be released from different heights by releasing a metal pin. (**C**) The released weight falls down the tube and simulates the mallet strike on the stem.

A custom-built metal tube and weights of 250 g each were placed vertically above the stem (Figure 4). The weight was dropped from fixed heights of 20 to 42.5 cm on the apex of the stem by releasing a metal pin from the tube. We used a gradually increasing protocol with 10 strikes per step. Five initial strikes from the smallest height simulated the entrance of the stem into the shaft. This aimed to simulate the initial cautious strikes by the surgeon. After the stem had reached its starting position within the humeral shaft, steps 2 to 5 simulated surgical mallet strikes with an incremental increase in energy. From step 2 to 3 and 3 to 4, the total energy in joules nearly doubled. Due to the repetitive protocol and the incremental increase in energy, one can calculate an exact energy until failure for every specimen. This is possible because the restoring force is not large enough to induce a backward movement of the stem (Table 2).

**Figure 5:**
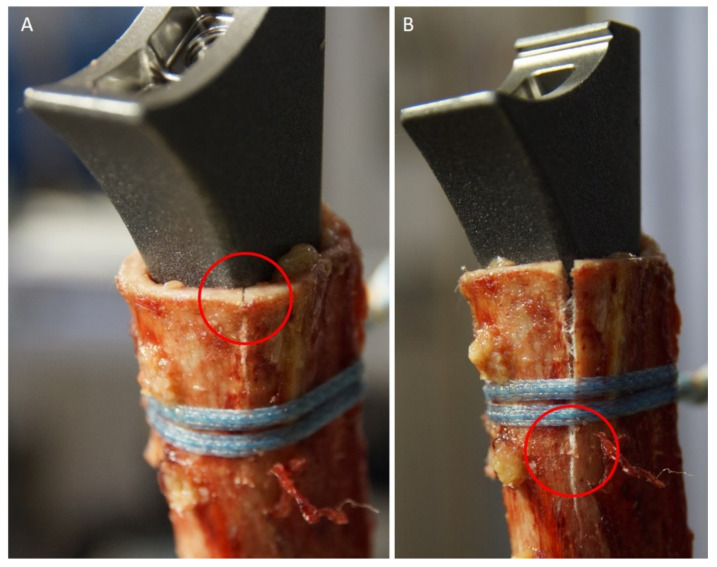
(**A**) First crack at the humeral shaft entrance. (**B**) Complete humeral shaft fracture progressing distally to the cerclage.

After every weight drop, the bone was inspected by 2 independent investigators for visible cracks. The first outcome parameter was defined as the total energy in joules (J) applied until the first crack (proximal to the cerclage) became visible. The second outcome parameter was defined as the total energy applied up until the occurrence of a full fracture of the humeral shaft, which had occurred distally to the cerclage (Figure 5). Additionally, the subsidence of the stem into the shaft (in mm) was documented.

### 2.5. Statistical Analysis

Statistical calculations were performed with GraphPad Prism 8 (San Diego, CA, USA). The parameters of intramedullary diameter, stem size and stem progression were analysed using multiple *t*-tests (unpaired *t*-test with Welch’s correction). Using the Bonferroni correction, we set significance at *p* < 0.017. The other outcome parameters were analysed using *t*-tests (unpaired *t*-test with Welch’s correction). Statistical significance was set at *p* < 0.05.

## 3. Results

All 28 humeri were biomechanically tested. However, one specimen (group FT-hand) was excluded from the statistical analysis. A retrospectively detected severe mismatch between shaft diameter and implanted stem in this specimen led to a disproportionally high energy until fracture, which had to be rated as a statistical outlier.

**Figure 6:**
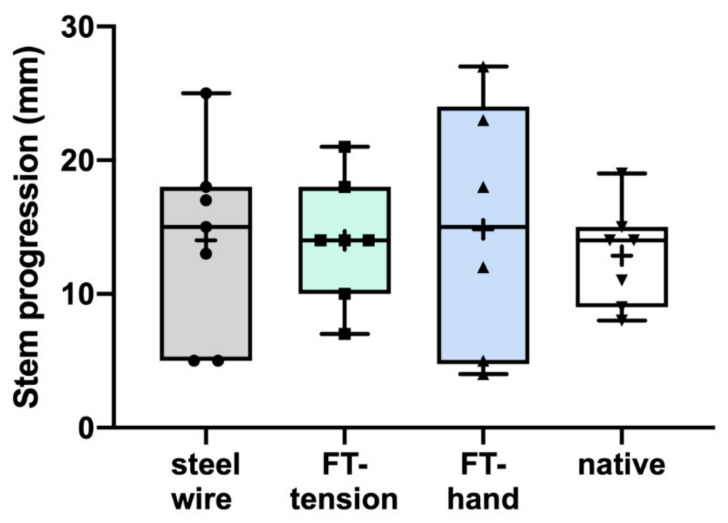
Stem progression measured before the start of the biomechanical testing to the appearance of a fracture did not differ between groups.

The mean intramedullary diameter at the humeral shaft entrance of the remaining 27 specimens measured 17.1 mm (range 13–23 mm). The median stem size was 8 mm (range 6–13 mm). There was no significant difference between groups regarding those values. The mean stem subsidence into the humeral shaft was 13.9 mm (range 5–27 mm) and did not differ between groups (Figure 6).

**Figure 7:**
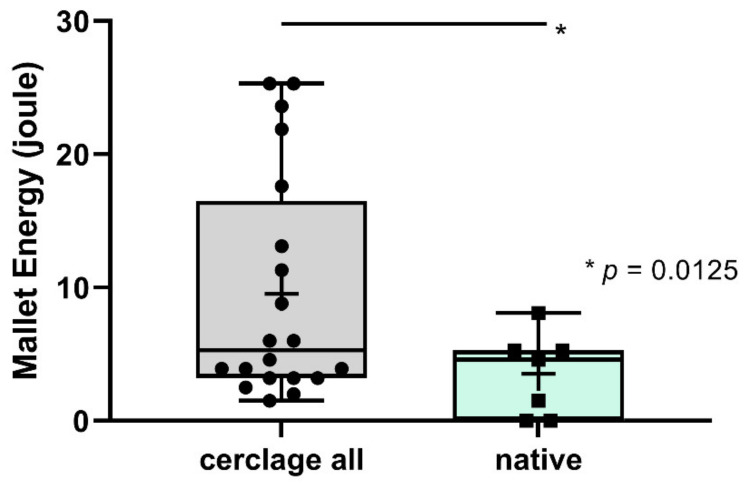
Mallet energy until occurrence of a full fracture was significantly higher for all cerclage groups (cerclage all) compared to the native situation (native).

The mallet energy applied until the occurrence of a first crack for all groups was 4.9 J (range 1–20.2 J). There was no significant difference between groups. The applied energy up until the occurrence of a full fracture of the humeral shaft differed significantly between all cerclage groups and the native group (9.5 J vs. 3.5 J, respectively; *p* = 0.0125) (Figure 7).

**Figure 8:**
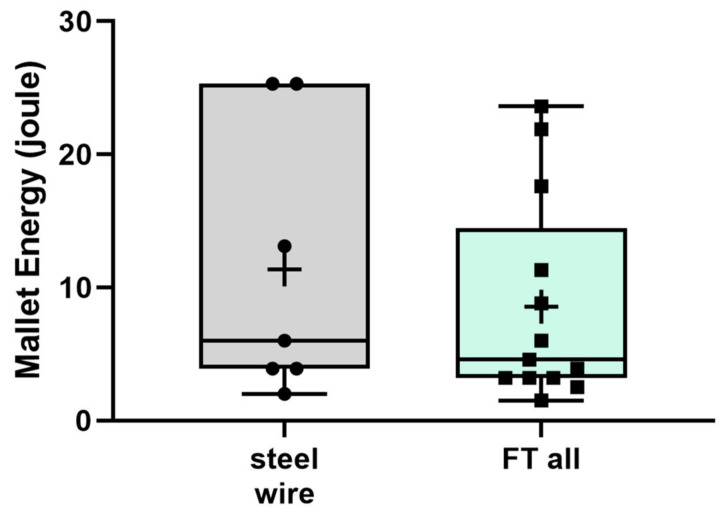
Mallet energy until occurrence of a full fracture was not different between the group steel wire cerclage (steel wire) and all FiberTape groups (FT all).

There was no difference between the steel wire and both FiberTape groups (11.4 J vs. 8.6 J, respectively; *p* = 0.2695) (Figure 8).

We detected no difference for the FiberTape hand group compared to the FiberTape tensioned group (10.2 J vs. 7.2 J, respectively; *p* = 0.2587). All fractures were located at the concave side of the stem (Figure 5).

## 4. Discussion

This biomechanical study demonstrates that a protective proximal humeral shaft cerclage decreases the risk of a periprosthetic fracture during implantation of RSA. The FiberTape cerclage is biomechanically equally efficient compared to a steel wire cerclage. The tensioning procedure (hand-tied vs. cerclage tensioner) does not influence the outcome.

To our knowledge, no biomechanical data are available on the protective effects of a humeral cerclage during implantation of RSA. Biomechanical studies have investigated the effect of a protective cerclage during hip arthroplasty. Several investigators found that prophylactic cerclages can prevent crack propagation and increase the rotational- and total energy to failure in press-fit femoral implants [23,24,33,34]. These studies created an osteotomy and used a biomechanical testing machine to apply a constant propulsion force on the femoral implant. This only reflects the clinical reality of shaft preparation and stem impaction partially. High peak forces during broaching or hammering on the stem during impaction can create a hoop-stress fracture [35,36]. We therefore aimed to develop a standardized biomechanical model and designed a new custom-built setup, which accurately simulates the volatile forces during the stem impaction process. In contrast to other studies, this enabled us to examine the total mallet energy in joules until humeral shaft failure. We defined the first endpoint as the energy applied up until the occurrence of a visible first crack. The second endpoint was the total energy used until fracture progression distally to the cerclage. For the native group, the energy until the first crack was equal to the energy until humeral shaft fracture. Without a cerclage to support the humeral shaft, the humeral shaft would fracture completely with the next mallet strike after the occurrence of a crack.

Another important aspect was the location of the fracture on the concave side of the stem (see Figure 5). With progression of the stem into the shaft, the stem overrides the metaphyseal bone, leading to fracture progression. While the classic Grammont prosthesis featured a straight stem with an inlay humeral tray, most newer prosthesis designs have a curved stem with an onlay humeral tray [37]. In revision surgery after failed PHF fixation, the humeral shaft is already weakened by the bi-cortical screw holes of the plate. Although we did not directly compare different stem designs in our study, we were able to demonstrate that a cerclage can be protective during the impaction of a curved designed stem.

Deeper analysis of the different cerclage materials did not show significant differences between the steel wire and FiberTape groups. This finding is in line with a biomechanical study from Renner et al. The authors investigated the properties of different cerclage materials with two models. A half-shell model with bovine femora showed significantly higher tightening strength for the steel cerclage wire (1.25 mm) compared to the FiberWire (No. 5) cerclage (817 ± 16 N vs. 131 ± 6 N, respectively). The periprosthetic fracture model showed similar resistance values (power in N, to move the prothesis 1 mm into the humeral shaft) for the cerclage wire and FiberWire cerclage technique. The authors used a double 1.25 mm steel cerclage wire and a fourfold FiberWire No. 5 [38]. The biomechanical advantage for the steel cerclage wire in the half-shell model diminished in the periprosthetic fracture model. In a clinical context, the FiberTape cerclage might even be advantageous due to a smaller complication rate regarding soft-tissue irrigation. Additionally, the tape cerclage is easier to handle for the surgeon and reduces the risk of injury during the application process [39].

A limitation of this study was the time-zero biomechanical testing, which cannot simulate the ongoing healing process in a regular patient. Although performed very carefully, the stem selection in relation to the corresponding humeral shaft was difficult and there was a chance of under- or over-estimation. This is reflected by the high range of stem progression (5–27 mm) into the humeral shaft. On the other hand, this simulated the clinical situation during the implantation process exactly. Another limitation was the low total number of specimens per group (7). This was due to the fact that human specimens are difficult to obtain. Additionally, the bone quality of all specimens was osteoporotic, which might not thoroughly be comparable to non-osteoporotic bone.

## 5. Conclusions

In conclusion, this study demonstrates that a protective cerclage can successfully delay the occurrence of a fracture during stem impaction in reverse shoulder arthroplasty for failed proximal humerus fracture fixation. A FiberTape cerclage is biomechanically equally efficient compared to a steel wire cerclage. The tensioning technique does not influence the biomechanical stability of the humerus. Especially for uncemented and calcar dependent arthroplasty, this might be clinically helpful to ensure adequate fixation and rotational stability.

## Figures and Tables

**Table 1:** Mean bone mineral density (BMD) and deltoid tuberosity index (DTI) over all groups.

Groups	Steel Wire	FT-Tension	FT-Hand	Native
BMD (mg/cm^3^)	75.6 ± 30.3	83.8 ± 24.7	90.0 ± 38.8	89.5 ± 32.2
DTI	1.32 ± 0.08	1.33 ± 0.1	1.33 ± 0.06	1.32 ± 0.1

**Table 2:** Incremental biomechanical test protocol simulating the mallet strikes during stem impaction.

	Step 1	Step 2	Step 3	Step 4	Step 5
Weight (g)	250	250	250	500	500
Height (cm)	20	27.5	35	35	42.5
Strikes	5	10	10	10	10
Joules per strike	0.5	0.7	0.9	1.7	2.1
Joules per step	2.5	7	9	17	21
Joules in total	2.5	9.5	18.5	35.5	56.5

## Data Availability

The data presented in this study are available on request from the corresponding author.
